# ADS024, a *Bacillus velezensis* strain, protects human colonic epithelial cells against *C. difficile* toxin-mediated apoptosis

**DOI:** 10.3389/fmicb.2022.1072534

**Published:** 2023-01-10

**Authors:** Ying Xie, Andrea Chupina Estrada, Becca Nelson, Hanping Feng, Charalabos Pothoulakis, Laurent Chesnel, Hon Wai Koon

**Affiliations:** ^1^Vatche and Tamar Manoukian Division of Digestive Diseases, David Geffen School of Medicine at the University of California Los Angeles, Los Angeles, CA, United States; ^2^Department of Gastroenterology, The First Hospital of China Medical University, Shenyang, Liaoning, China; ^3^Department of Microbial Pathogenesis, School of Dentistry, University of Maryland, College Park, College Park, MD, United States; ^4^Adiso Therapeutics Inc., Concord, MA, United States

**Keywords:** infection, biologic, therapy, single-strain live biotherapeutic product, apoptosis, *C. difficile*

## Abstract

*Clostridioides difficile* infection (CDI) causes intestinal injury. Toxin A and toxin B cause intestinal injury by inducing colonic epithelial cell apoptosis. ADS024 is a *Bacillus velezensis* strain in development as a single-strain live biotherapeutic product (SS-LBP) to prevent the recurrence of CDI following the completion of standard antibiotic treatment. We evaluated the protective effects of the sterile filtrate and ethyl acetate extract of conditioned media from ADS024 and DSM7 (control strain) against mucosal epithelial injury in toxin-treated human colonic tissues and apoptosis in toxin-treated human colonic epithelial cells. Ethyl acetate extracts were generated from conditioned culture media from DSM7 and ADS024. Toxin A and toxin B exposure caused epithelial injury in fresh human colonic explants. The sterile filtrate of ADS024, but not DSM7, prevented toxin B-mediated epithelial injury in fresh human colonic explants. Both sterile filtrate and ethyl acetate extract of ADS024 prevented toxin-mediated apoptosis in human colonic epithelial cells. The anti-apoptotic effects of ADS024 filtrate and ethyl acetate extract were dependent on the inhibition of caspase 3 cleavage. The sterile filtrate, but not ethyl acetate extract, of ADS024 partially degraded toxin B. ADS024 inhibits toxin B-mediated apoptosis in human colonic epithelial cells and colonic explants.

## Introduction

*Clostridioides difficile* infection (CDI) is common among patients with prolonged antibiotic exposure (Marra and Ng, 2015; Reveles et al., 2017). Toxigenic *C. difficile* produces toxin A and toxin B that mediate cell death and inflammatory responses (Kuehne et al., 2010; Shen, 2012). Clinically, infected patients present with diarrhea, bloody stool, and abdominal pain. Many patients can be cured with vancomycin or fidaxomicin (Cornely et al., 2012, 2014; Crook et al., 2012; McDonald et al., 2018; Johnson et al., 2021). Even though a clinical cure can be achieved with standard-of-care antibiotics, the risk of intestinal microbiota disruption is exceedingly high and further contributes to the recurrence of CDI. This complication created the need for secondary therapies to prevent recurrences, such as anti-toxin B monoclonal neutralizing antibody (Bezlotoxumab) and fecal microbiota transplantation. Injection of Bezlotoxumab mildly reduced the CDI recurrence rate (Hengel et al., 2020). Although fecal microbiota transplantation has shown a 90% success rate (Borgia et al., 2015), additional efforts are needed to address safety concerns and other technical improvements (Wang et al., 2016; Shin et al., 2018; DeFilipp et al., 2019). New preventive and therapeutic approaches are currently being developed as live biotherapeutic products (LBPs), including numerous capsule-based standardized microbiota restoration approaches (Feuerstadt et al., 2021; Khanna et al., 2021).

*Bacillus amyloliquefaciens* is a bacterial species that inhibits CDI colitis in mice by inhibiting the bacterial growth of *C. difficile* VPI10463 (Geeraerts et al., 2015). Importantly, oral administration of a *B. amyloliquefaciens* culture showed better therapeutic efficacy than the *Saccharomyces boulardii* culture in lowering colonic toxin levels and reducing colonic injury in infected mice (Geeraerts et al., 2015). The genome of *Bacillus amyloliquefaciens* FZB42 dedicates 8.5% of its genome to antimicrobial peptide production (Chen et al., 2007). For example, *B. amyloliquefaciens* C-1 carries antimicrobial gene clusters of surfactin, iturins, fengycins, and others (Lv et al., 2020).

ADS024 is a single-strain live biotherapeutic product (SS-LBP) of *Bacillus velezensis*, considered a member of the “operational group *B. amyloliquefaciens.*” This group comprises the soil-borne *B. amyloliquefaciens* and plant-associated *Bacillus siamensis* and *Bacillus velezensis*, which is synonymous with *B. amyloliquefaciens* subsp. plantarum (Fan et al., 2017). ADS024 was identified from a large-scale screening of stool samples to detect aerobic spore-forming bacteria with direct anti-*C. difficile* activity and proteolytic capability (O’Donnell et al., 2022). ADS024 is being investigated as a single-strain live biotherapeutic product (SS-LBP) candidate to prevent CDI recurrence following successful antibacterial treatment. SS-LBPs are uniquely identified and differentiated from consortia LBPs as biological medicinal products containing live microorganism(s) as active substance(s) and can be used for prevention, treatment, or cure of disease (Rouanet et al., 2020). LBPs can modulate the host and its microbiota through various mechanisms of action, including suppression of pathogens, balancing mucosal immune system activity through a reduction in the inflammatory response, and activating cellular pathways within cells (Rouanet et al., 2020).

Since intestinal epithelial cell death can alter the gut barrier (Patankar and Becker, 2020), we focused on studying whether ADS024 can prevent *C. difficile* toxin-mediated cell death and apoptosis. We hypothesize that ADS024 produces and secretes enzymes and metabolites that protect colonic epithelial cells against *C. difficile* toxins. Therefore, we evaluated the cytoprotective effects of ADS024 in toxin-treated human colonic explants and epithelial cells.

## Materials and methods

### Bacterial preparations

Lyophilized ADS024 and control strain *B. amyloliquefaciens* DSM7 were used. A single lot of lyophilized material was used in all experiments assuring consistency throughout all studies. The bacterial stock was dissolved to 6 × 10^9^ colony forming units (cfu)/ml in RPMI1640 (pH 8) and incubated at 37°C for 30 min before use. The bacterial suspensions were filtered with 0.22 μm filters to produce sterile filtrates, which were further serially diluted to 1X, 0.1X, 0.01X, 0.0001X, and 10^–6^X in PBS (pH 7.4). 1 μL of the sterile filtrates were added to cells and tissue cultures (1 ml) to final titers of 6 × 10^6^ cfu/ml, 6 × 10^5^ cfu/ml, 6 × 10^4^ cfu/ml, 6 × 10^2^ cfu/ml, and 0.6 cfu/ml. The bacterial filtrates were used within 1 h of resuspension to ensure freshness. Sterile filtrates of bacteria represent the metabolites, proteins, or extracellular vesicles secreted by the live bacteria during the 30-min rehydration period. The use of sterile filtrates prevented bacterial contamination of the cell culture incubator.

As described previously, isopropanol (IPA) extracts were prepared from the centrifuged bacterial cells (O’Donnell et al., 2022). IPA extracts are enriched in membrane-bound and internal bacterial metabolites. Ethyl acetate (EA) extracts from the bacteria-conditioned media of ADS024 and DSM7 were prepared by Adiso Therapeutics as described previously (O’Donnell et al., 2022) and represent enriched samples of bacteria-secreted metabolites. The media without bacteria were made into the control EA extract. PBS was used as a control for IPA extract groups.

The IPA and EA lyophilized extracts were weighed and dissolved in PBS solution to generate 100 mg/ml stocks. These stocks were further diluted to 1X, 0.1X, 0.01X, and 0.001X in PBS. Finally, 10 μL of diluted stocks were added to the cell or tissue cultures (1 ml) to represent final concentrations of 1,000 μg/ml, 100 μg/ml, 10 μg/ml, and 1 μg/ml, respectively.

The use of ADS024 filtrate was to determine the effects of all secreted products of ADS024, including metabolites, proteins, and enzymes while avoiding any solvents that might denature secreted membrane vesicles or proteins. ADS024 ethyl acetate was used to detect the effects of secreted metabolites in the ADS024-conditioned media. The proteins and enzymes were inactivated during the ethyl acetate extraction process. ADS024 isopropanol extract was used to determine the effects of membrane-bound and intracellular metabolites in the ADS024 bacteria by removing denatured large macromolecules during extraction.

### Human colonic explant preparation

Fresh human colonic explants were isolated from non-cancerous regions of ten colon cancer patients (UCLA Surgical Pathology), as described previously (Koon et al., 2013, 2017; Wang et al., 2019). In short, the fresh colonic explants were cut into 3 × 3 mm sizes, placed in serum-free RPMI1640 medium, and incubated at 37*^o^*C for 30 min, followed by the addition of ADS024 and DSM7 sterile filtrates, IPA extracts, or EA extracts. Thirty minutes later, the tissues were treated with PBS, toxin A (0.1 μg/ml), or toxin B (0.1 μg/ml) and incubated at 37°C. After 6 h of incubation, half of the conditioned media were taken for Duo-Set ELISAs, including TNFα (DY210), IL-1β (DY201), and MIP-1α (DY270) from R&D Systems. After 24 h of incubation, the tissues were taken for histological evaluation. The baseline characteristics of the patients are shown in Supplementary Table 1.

### Ethics statement

Institutional Review Board (IRB) approved the human colonic explant sample study (IRB 12-001499). No patient-identifiable information was obtained. In addition, informed consent was waived by UCLA IRB.

### Histology scoring

The treated fresh human colonic tissues were fixed in formalin solution overnight. The fixed tissues were paraffin-embedded, cut at 4 μm thickness, and stained with hematoxylin and eosin (H&E) at the UCLA Tissue Processing Core Laboratory (TPCL). Two independent observers blindly evaluated the stained slides by observing four different mucosal locations per tissue section. The severity of colonic tissue injury was graded for epithelial tissue damage on a scale of 0–3 (Pothoulakis et al., 1994).

### Immunofluorescence staining of fresh human colonic explants

The TPCL performed tri-color immunofluorescence staining of the treated fresh colonic tissues. Briefly, paraffin on the sections was removed with xylene, and the tissue sections were rehydrated through graded ethanol. Endogenous peroxidase activity was blocked with 3% hydrogen peroxide in methanol for 10 min. Next, heat-induced antigen retrieval (HIER) was carried out for all sections in 0.01 M citrate buffer, pH = 6, using a Biocare decloaker at 95°C for 25 min. After treatment with blocking buffer, pH 7.4, for 1 h, the slides were incubated overnight at 4°C with primary antibodies to cleaved caspase 3 (#9661, cell signaling, Danvers, MA, United States) and pan-cytokeratin (M3515, Dako) in 2% BSA at 1:100 dilution. Anti-rabbit (red) and anti-mouse (green) secondary antibodies and DAPI (blue) nuclear stain were added on day 2. These multiplex reagents were included in the OPAL staining kit. The entire tissue sections were scanned by the Leica Aperio Versa system and analyzed by Phenochart software. Images of mucosal regions were captured based on mucosal structures and green epithelial cell signals. Pixels of images in red, green, and blue channels were shown on the histograms in Adobe Photoshop. The signals of red (cleaved caspase 3) and green (pan-cytokeratin) were first normalized to the blue (nuclear) signal, and the red/green ratios were calculated in percentage.

### Human colonic epithelial cells

Human colonic epithelial NCM460 cells were cultured in an M3D medium (Incell, San Antonio, TX, United States) (Koon et al., 2017). Primary human colonic epithelial cells or HPEC (H6047, Cell Biologics, Chicago, IL, United States) were cultured in a medium (H6621 medium, Cell Biologics, Chicago, IL, United States). All cells were grown in media containing 10% fetal bovine serum and 1% penicillin-streptomycin to 80% confluence and then switched to serum-free media overnight.

Cells were starved of serum overnight and then were pretreated with 1 μL/ml sterile filtrates or 10 μL/ml IPA/EA extracts for 30 min, followed by toxin A and toxin B addition at 0.1 μg/ml and further incubated at 37*^o^*C for 6–24 h. The production of toxin A and toxin B were described in our previous report (Yang et al., 2008). Our previous studies determined that toxin A and toxin B at 0.1 μg/ml were optimal for inducing pro-inflammatory cytokine secretion and mucosal damage in fresh human colonic tissues (Koon et al., 2017; Wang et al., 2019).

For apoptosis determination, the reagents of RealTime-Glo Annexin V apoptosis assay (JA1011, Promega, Madison, WI, United States) were added to the white-wall clear-bottom 96-well plates at 0 h. The luminescence (apoptosis) signal was measured with a BioTek Synergy H1 plate reader.

For apoptosis-related protein discovery, serum-starved HPECs in 6-well plates were pretreated with ADS024 ethyl acetate extract at 1X or sterile filtrate at 0.0001X dilution for 30 min, followed by toxin B (0.1 μg/ml) for 24 h. The cell lysates (300 μg protein/group) were collected for Proteome Profiler Human Apoptosis Array Kit (ARY009, R&D Systems, Minneapolis, MN, United States). All reagents used were included in the kit. Signals on the protein arrays were detected by a Bio-Rad ChemiDoc Imaging system and quantified by Bio-Rad Image Lab software.

For cleaved caspase 3 measurement by ELISA, the serum-starved cells were incubated with toxins and sterile filtrates or IPA/EA extracts for 24 h and then lyzed by radioimmunoprecipitation assay (RIPA) buffer, pH 7.5 (#89900, ThermoFisher, Waltham, MA, United States) containing a 1X protease inhibitor cocktail (#78429, ThermoFisher, Waltham, MA, United States). The same protease inhibitor cocktail was also applied to stop protease activity in the NCM460 apoptosis experiment. Cleaved caspase 3 in the lysates was determined by ELISA (KHO1091, ThermoFisher, Waltham, MA, United States). Protein concentrations in cell lysates were determined by a bicinchoninic acid (BCA) assay (#23225, ThermoFisher, Waltham, MA, United States).

The serum-starved NCM460 cells were incubated with sterile filtrates or IPA/EA extracts for 48 h to determine cell viability. MTS-based CellTiter 96 aqueous non-radioactive cell proliferation assay (G5421, Promega, Madison, WI, United States) reagent (5 μl/100 μL) was added to the cell culture media and incubated for 15 min (Koon et al., 2007). The colorimetric signal at 490 nm was measured with a BioTek Synergy H1 plate reader.

### Toxin digestion assays

Sterile filtrates of ADS024 and DSM7 were diluted from 1X to 10^–6^X in PBS. IPA/EA extracts of ADS024 and DSM7 were added as 1X. For molecular weight cut-off (MWCO) testing, the ADS024 filtrate was filtered by Vivaspin 2 MWCO columns (100 kDa: 45-001-570; 50 kDa: 45-001-569; 30 kDa: 45-001-568; 10 kDa: 45-001-567; 5 kDa: 45-001-566; 3 kDa: 45-001-565) from (Cytiva, Marlborough, MA, United States). The filtrates and extracts were incubated with 50 ng toxin A or toxin B in 500 μL serum-free RPMI1640 media at 37°C for 1 h. The digestion was then stopped by a protease inhibitor cocktail (PIC) and ethylenediaminetetraacetic acid (EDTA) (#78446, ThermoFisher, Waltham, MA, United States) at 1X. The mixtures were used for toxin level determination by ELISA (ABIN1098189, antibodies-online.com, Limerick, PA, United States).

### Statistical analysis

All experiments were repeated to ensure reproducibility. Unpaired Student’s *t*-tests were used for two-group comparisons of continuous data, and ordinary one-way ANOVAs were used for multiple-group comparisons (GraphPad Prism). Results were expressed as mean ± standard deviation. Significant *p*-values are shown in each figure.

### Data availability statement

We may share additional unpublished data from the study. Please contact Hon Wai Koon or Adiso Therapeutics. The Adiso’s company website is: https://adisotx.com/

## Results

*ADS024 filtrate and ethyl acetate extracts prevented C. difficile toxin-mediated apoptosis.*

Although many operational Group *B. amyloliquefaciens* strains are known to produce antimicrobial compounds (Lv et al., 2020; Gerst et al., 2021), we sought to discover the direct protective effects of ADS024 and DSM7 in toxin-treated human colonic epithelial cells. *C. difficile* toxins kill human colonic epithelial cells *via* apoptosis (Fiorentini et al., 1998; Brito et al., 2002), leading to intestinal injury.

**FIGURE 1:**
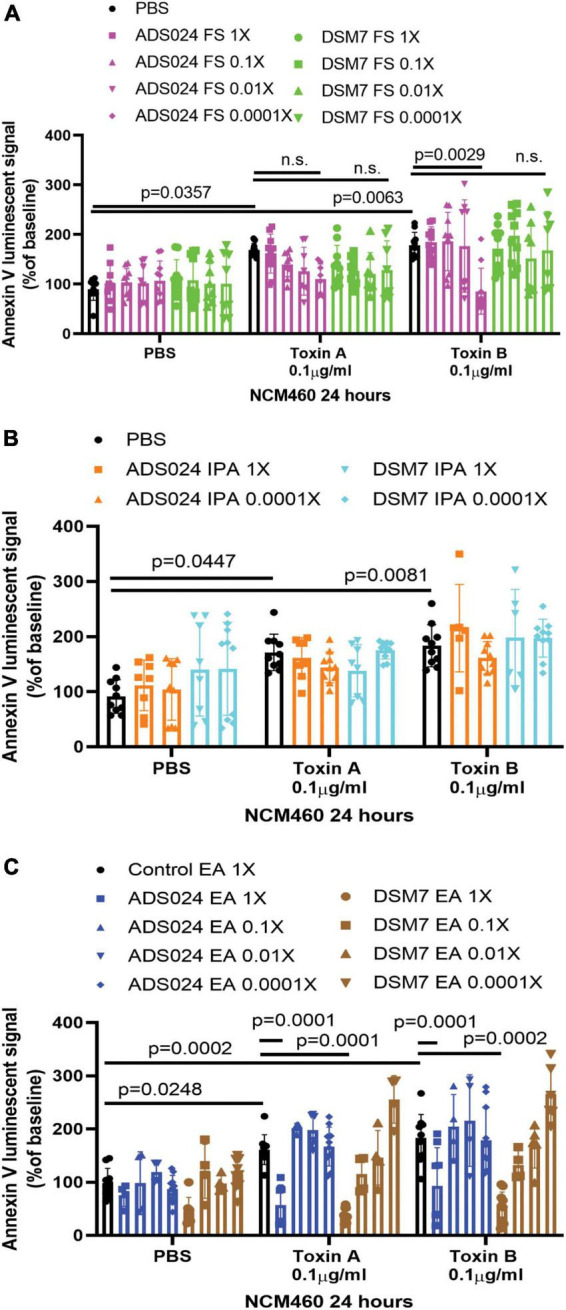
ADS024 sterile filtrate and ethyl acetate extract possess anti-apoptotic effects in human colonic epithelial cells. **(A–C)** Apoptosis assays of human colonic epithelial NCM460 cells. The Annexin V luminescent signal above 100% indicated the occurrence of apoptosis. **(A)** Serum-starved NCM460 cells were treated with PBS or 0.1% v/v of ADS024 and DSM7 sterile filtrate (FS) from 1 to 0.0001-fold. **(B)** Serum-starved NCM460 cells were treated with PBS or 1% v/v of ADS024 and DSM7 isopropanol extract (IPA) from 1 to 0.0001-fold. **(C)** Serum-starved NCM460 cells were treated with PBS or 1% v/v of ADS024 and DSM7 ethyl acetate extract (EA) from 1- to 0.0001-fold. **(A–C)** After adding sterile filtrates (FS), IPA extracts, and EA extracts, the cells were added with Promega RealTime-Glo Annexin V apoptosis assay reagents in a 1:1,000 ratio. Thirty minutes later, the cells were added with PBS, toxin A (0.1 μg/ml), or toxin B (0.1 μg/ml). After 24 h, the luminescent (apoptosis) signals were read by a 96-plate reader. Toxins increased the apoptosis signal. ADS024 FS at 0.0001X reduced toxin B-induced apoptosis. EA extracts of ADS024 and DSM7 at 1X reduced toxin A- and B-induced apoptosis. The results were pooled from 3–4 independent experiments. One-way ANOVA tests were used.

Both toxin A and toxin B induced apoptosis in human colonic epithelial cells (Figure 1A). The ADS024, but not DSM7, filtrate at 0.0001X prevented toxin B-mediated apoptosis (Figure 1A). However, ADS024 and DSM7 filtrates and IPA extracts failed to prevent toxin A-mediated apoptosis (Figures 1A, B). Notably, EA extracts of ADS024 and DSM7 conditioned media at 1X effectively prevented toxin A- and B-mediated apoptosis in human colonic epithelial cells (Figure 1C). As ADS024 filtrate and EA extract prevented apoptosis in toxin B-treated human colonic epithelial cells, the bacteria secrete active protective agents.

*ADS024 filtrate prevented toxin B-mediated epithelial injury in human colonic explants.*

**FIGURE 2:**
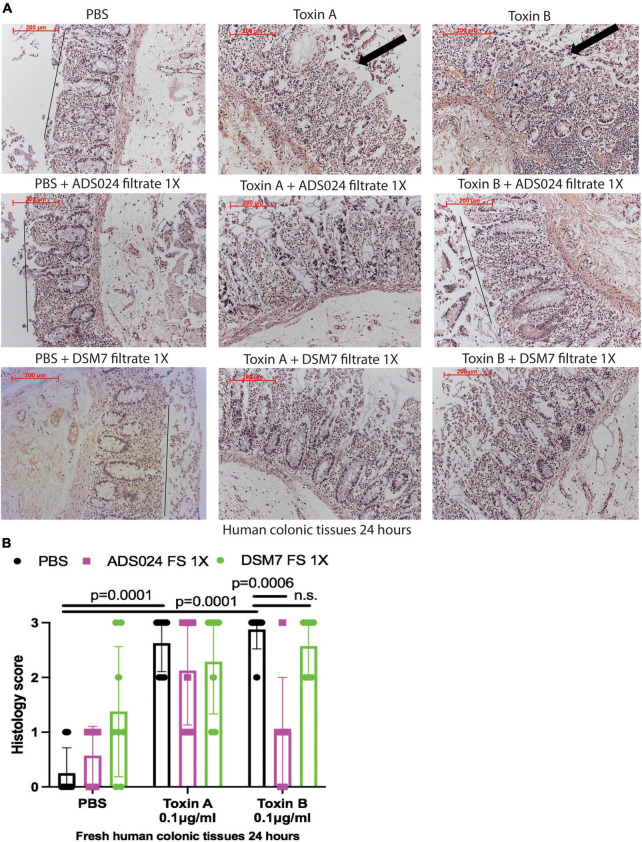
ADS024 sterile filtrate reduced toxin B-mediated epithelial injury in fresh human colonic explants. Fresh human colonic explants were placed in serum-free RPMI1640 media with PBS or 0.1% v/v of ADS024 and DSM7 sterile filtrate (FS) at 1X. Thirty minutes later, toxin A and toxin B (0.1 μg/ml) were added and further incubated for 24 h. **(A)** H&E-stained images at 100X magnification. Black lines indicated the integrity of the top lining of colonic mucosa in PBS control and toxin B- and ADS024 filtrate-treated groups. Arrows indicated toxin-mediated disruption of mucosal integrity and thickness. **(B)** Histology scores for epithelial injury (0–3). ADS024 sterile filtrate 1X reduced toxin-B mediated epithelial injury. Results were pooled from samples from 10 patients. One-way ANOVA tests were used.

To evaluate the protective effects of ADS024 and DSM7 metabolites, we pretreated fresh human colonic explants with ADS024 and DSM7 filtrates at 1X, followed by toxins exposure. Consistent with the protective effects of ADS024 filtrate in toxin B-treated colonic epithelial cells (Figure 1A), ADS024 filtrate at 1X prevented toxin B-, but not toxin A-, mediated epithelial injury in fresh human colonic explants (Figures 2A, B). On the other hand, DSM7 filtrate at 1X failed to affect epithelial injury caused by both toxins (Figures 2A, B).

*ADS024 and DSM7 ethyl acetate extracts prevented toxin-mediated epithelial injury in human colonic explants.*

**FIGURE 3:**
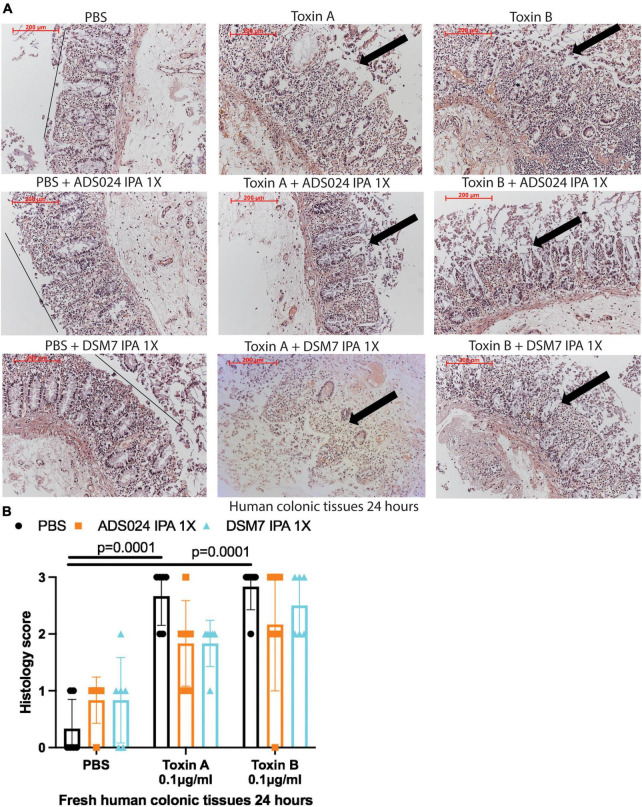
ADS024 isopropanol extract did not affect toxin-mediated epithelial injury in fresh human colonic explants. Fresh human colonic explants were placed in serum-free RPMI1640 media with PBS or 0.1% v/v of IPA extracts of ADS024 and DSM7 at 1X. Thirty minutes later, toxin A and toxin B (0.1 μg/ml) were added and further incubated for 24 h. **(A)** H&E-stained images at 100X magnification. Black lines indicated the integrity of the top lining of colonic mucosa in PBS control groups. Arrows indicated toxin-mediated disruption of mucosal integrity and thickness. **(B)** Histology scores for epithelial injury (0–3). ADS024 and DSM7 IPA extracts did not affect toxin-mediated epithelial injury. Results were pooled from samples from 10 patients. One-way ANOVA tests were used.

**FIGURE 4:**
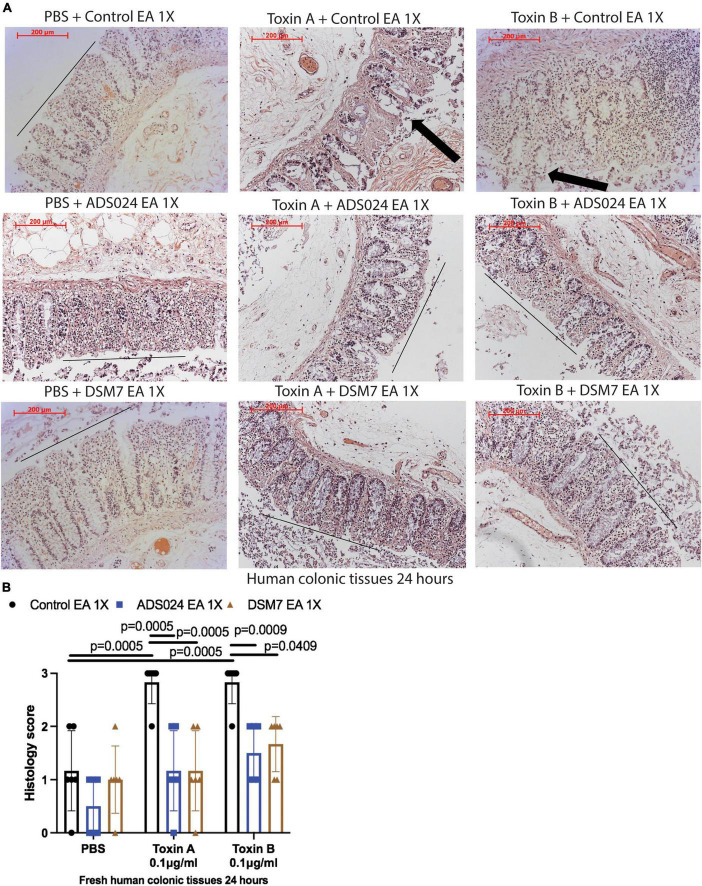
ADS024 ethyl acetate extract reduced toxin-mediated epithelial injury in fresh human colonic explants. Fresh human colonic explants were placed in serum-free RPMI1640 media with PBS or 0.1% v/v of ADS024 and DSM7 EA extracts at 1X. Thirty minutes later, toxin A and toxin B (0.1 μg/ml) were added and further incubated for 24 h. **(A)** H&E-stained images at 100X magnification. Black lines indicated the integrity of the top lining of colonic mucosa in PBS control groups and toxin- and EA extract-treated groups. Arrows indicated toxin-mediated disruption of mucosal integrity and thickness. **(B)** Histology scores for epithelial injury (0–3). EA extracts of ADS024 and DSM7 reduced toxin-mediated epithelial injury. Results were pooled from samples from 10 patients. One-way ANOVA tests were used.

IPA extracts of ADS024 and DSM7 did not affect toxin-mediated epithelial injury in fresh human colonic explants (Figures 3A, B). On the other hand, consistent with the protective effects of ADS024 and DSM7 EA extracts in colonic epithelial cells (Figure 1C), EA extracts of both bacterial strains significantly reduced toxin A- and toxin B-mediated epithelial injury in fresh human colonic explants (Figures 4A, B).

*ADS024 filtrate partially digested toxin B*.

**FIGURE 5:**
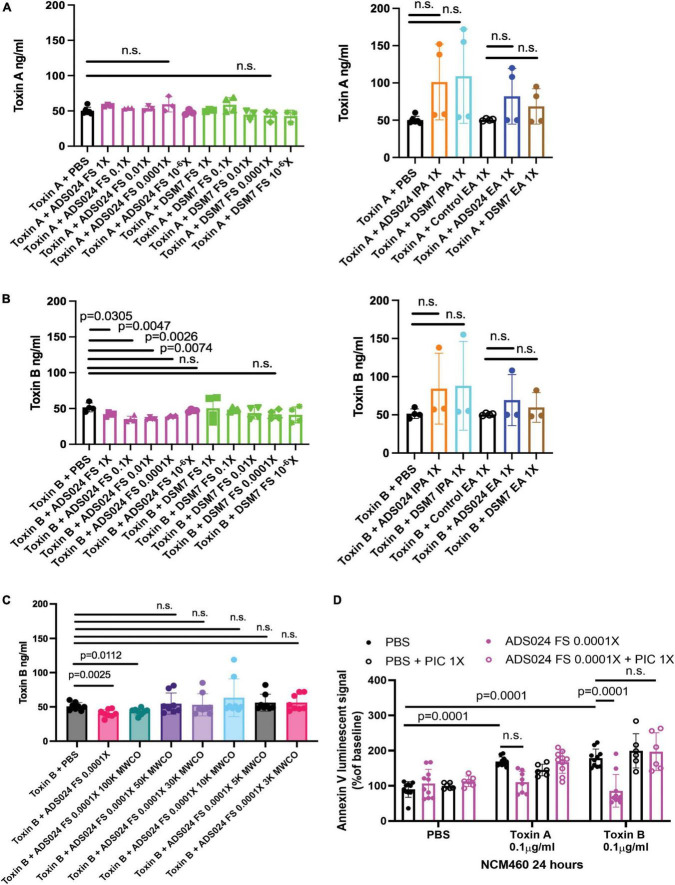
Protease inhibitors abolished the anti-apoptotic effect of ADS024 sterile filtrate. **(A–C)** Toxin digestion assays. Sterile filtrates, IPA extracts, and EA extracts of ADS024 and DSM7 from 1X to 10^– 6^X were added to serum-free RPMI1640 containing 50 ng/500 μl toxin A or toxin B. **(C)** ADS024 filtrates were further processed by MWCO columns. The centrifuged eluates were diluted to 0.0001X and added to serum-free RPMI1640 containing 50 ng/500 μl toxin B. **(A–C)** The mixtures were incubated at 37°C for 1 h and then stopped by adding a PIC and EDTA mixture (#PI78429, Thermo Scientific) in 1:100 dilution ratio. The toxin levels in the mixtures were measured by ELISA. ADS024 FS at 1–0.0001X partially degraded toxin B. MWCO at 50kDa abolished this effect. **(D)** Apoptosis assay. Serum-starved NCM460 cells were pretreated with ADS024 sterile filtrate at 0.0001X with or without protease inhibitor cocktail at 1X. Reagents of Promega RealTime-Glo Annexin V apoptosis assay in a 1:1,000 ratio were added simultaneously. Thirty minutes later, toxin A or toxin B (0.1 μg/ml) was added to start the apoptosis process. After 24 h, the luminescent (apoptosis) signals were read by a 96-plate reader. The Annexin V luminescent signal above 100% indicated the occurrence of apoptosis. The ADS024 sterile filtrate-mediated inhibition of apoptosis in toxin B-treated cells was reversed by pretreatment of PIC at 1X. Results were pooled from 3–4 independent experiments. One-way ANOVA tests were used.

Some operational group *B. amyloliquefaciens* strains produce proteases (Cho et al., 2003; Devaraj et al., 2018). ADS024 also possesses toxin-degrading activities (O’Donnell et al., 2022). Therefore, we attempted to characterize the toxin-degrading effects of ADS024 filtrates. ADS024 and DSM7 filtrates failed to degrade toxin A (Figure 5A). Conversely, incubation of toxin B with ADS024, but not DSM7, filtrates at 1–0.0001X partially reduced toxin B levels after 1-h incubation (Figure 5B). However, both IPA and EA extracts of ADS024 and DSM7 at 1X failed to degrade either toxin (Figures 5A, B, right panel).

To estimate the molecular weight of the potential toxin B-degrading agent in the ADS024 filtrate, we filtered the ADS024 filtrates with MWCO columns, followed by incubation with toxin B. MWCO at 100 kDa did not affect the toxin B-degrading efficacy of ADS024 filtrate. However, this toxin B-degrading effect was removed at MWCO from 50 kDa to 3 kDa (Figure 3C).

When human colonic epithelial cells were pretreated with a protease inhibitor cocktail (PIC), the ADS024 filtrate failed to exert anti-apoptotic effects against toxin B (Figure 5D). This result suggested that the anti-apoptotic effect of ADS024 filtrate depends on protease-mediated toxin B degradation.

*ADS024 prevented toxin-mediated apoptosis by inhibiting caspase 3 cleavage in colonic epithelial cells.*

**FIGURE 6:**
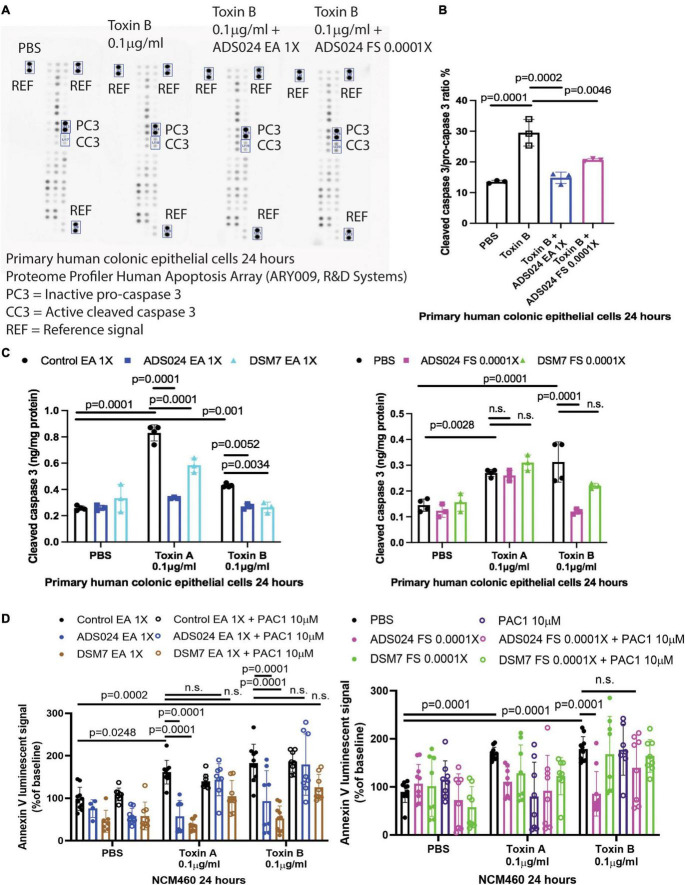
ADS024 inhibited caspase three cleavage in toxin-treated human colonic epithelial cells. **(A,B)** Apoptosis array. Serum-starved primary human colonic epithelial cells were pretreated with ADS024 ethyl acetate extract at 1X or sterile filtrate at 0.0001X dilution for 30 min, followed by toxin B (0.1 μg/ml). A total of 24 h later, the cells were collected for Proteome Profiler Human Apoptosis Array (ARY009, R&D Systems). **(A)** Bio-Rad ChemiDoc Imaging system captured the images. The rectangles highlighted the inactive procaspase three and active cleaved caspase three. The images are representative of three independent experiments. **(B)** Bio-Rad Image Lab Software performed quantitation of cleaved caspase 3/procaspase 3 signal. Toxin B increased cleaved caspase 3/pro-caspase 3 ratio, which was reduced by ADS024 EA extract 1X and sterile filtrate 0.0001X dilution. **(C)** Cleaved caspase 3 ELISA. Serum-starved primary human colonic epithelial cells were pretreated with ethyl acetate extracts of ADS024 and DSM7 at 1X or sterile filtrates of ADS024 and DSM7 at 0.0001X dilution for 30 min, followed by the addition of toxin B (0.1 μg/ml). The cells were lysed by RIPA buffer with 1X PIC and 1X EDTA. The cleaved caspase three levels in cell lysates were measured by ELISA. **(D)** Apoptosis assays. Serum-starved NCM460 cells were pretreated with ethyl acetate extracts of ADS024 and DSM7 at 1X and sterile filtrates of ADS024 and DSM7 at 0.0001X dilution with or without adding 10 μM procaspase activating compound 1 or PAC1 (#10009317, Cayman Chemical). PAC1 was used to activate caspase three cleavage. Reagents of Promega RealTime-Glo Annexin V apoptosis assay in a 1:1,000 ratio were added simultaneously. Thirty minutes later, toxin A or toxin B (0.1 μg/ml) was added to start the apoptosis process. After 24 h, the luminescent (apoptosis) signals were read by a 96-plate reader. The Annexin V luminescent signal above 100% indicated the occurrence of apoptosis. The ADS024 EA extract-mediated inhibition of toxin A- and B-dependent apoptosis was reversed by PAC1 pretreatment. The ADS024 sterile filtrate-mediated inhibition of apoptosis in toxin B-treated cells was reversed by PAC1 pretreatment. Results were pooled from 3–4 independent experiments. One-way ANOVA tests were used.

To discover a ADS024-associated anti-apoptotic pathway, we utilized a protein array to quantitatively detect the protein levels of 35 anti-/pro-apoptotic proteins in toxin B-treated HPEC simultaneously. Toxin B increased activated pro-apoptotic cleaved caspase 3 protein expression (Figures 6A, B), which was prevented by pretreatment with ADS024 EA at 1X and ADS024 filtrate at 0.0001X (Figure 6B).

An additional validation study by ELISA found that EA extracts of ADS024 and DSM7 significantly lowered cleaved caspase 3 protein expression in toxin-treated human colonic epithelial cells (Figure 6C). In addition, ADS024, but not DSM7, filtrate at 0.0001X significantly reduced toxin B-mediated caspase 3 cleavage (Figure 6C).

The addition of a caspase 3 activator (PAC1) attenuated the anti-apoptotic effects of EA extracts of ADS024 and DSM7 against toxin A and toxin B (Figure 6D). Similarly, PAC1 also abolished the anti-apoptotic effect of ADS024 filtrate against toxin B (Figure 6D). These findings suggested that ADS024 and DSM7 EA extracts and ADS024 filtrate exert anti-apoptotic effects against toxins *via* inhibition of caspase 3 cleavage in human colonic epithelial cells.

*ADS024 filtrate and ethyl acetate extract inhibited cleaved caspase 3 expression in toxin-treated human colonic explants.*

**FIGURE 7:**
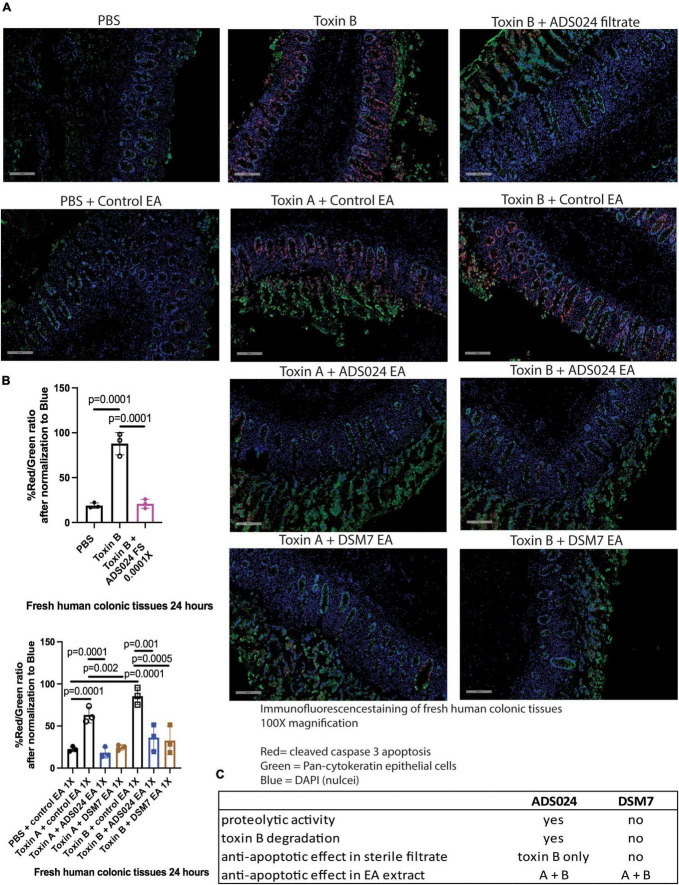
ADS024 inhibited caspase three cleavage in toxin-treated human colonic explants. **(A)** Immunofluorescence staining of cleaved caspase 3 (red), pan-cytokeratin (green), and nuclei (blue) in fresh human colonic explants treated with or without toxin A, toxin B, PBS, ADS024 filtrate, control EA, ADS024 EA, or DSM7 EA. The gray bars in the lower-left corner indicate 200 microns. Apoptosis (red, cleaved caspase) occurred in colonic epithelial cells (green, pan-cytokeratin) as red and green signals either overlapped or in proximity. **(B)** Quantitative analysis of images representing cleaved caspase three (red) and epithelial cells (green) ratio after normalized to blue nuclear signal. The high red/green ratio represented increased apoptosis. Toxin B-mediated cleaved caspase three was reduced by ADS024 filtrate treatment. Toxin A- and toxin B-mediated caspase three cleavages were reduced by ADS024 and DSM7 EA extract treatment. Results were representative of samples from 10 patients. One-way ANOVA tests were used. **(C)** A comparison of ADS024 and DSM7 properties.

Immunofluorescence staining showed that toxin A and toxin B increased cleaved caspase 3 protein expression in the epithelial cells of fresh human colonic mucosa (Figure 7). ADS024 filtrate reduced cleaved caspase 3 expression in toxin B-treated human colonic tissues (Figure 7A). In addition, ADS024 and DSM7 EA extracts reduced cleaved caspase 3 expression in toxin A- and B-treated fresh human colonic explants (Figure 7A). Quantitative image analysis indicated that the ADS024 filtrate and EA extract abolished toxin-induced cleaved caspase 3 expression (Figure 7B).

*ADS024 and DSM7 metabolites did not affect toxin-mediated proinflammatory cytokine secretion.*

Tumor necrosis factor-alpha (TNFα), interleukin-1 beta (IL-1β), and macrophage inflammatory protein-1 alpha (MIP-1α) were identified to be important in CDI disease development (McDermott et al., 2015; Yu et al., 2017; Wang et al., 2019). Consistent with our previous study (Wang et al., 2019), toxin A and toxin B induced TNFα, IL-1β, and MIP-1α secretion in fresh human colonic explants (Supplementary Figures 1A–C). However, filtrates, IPA extracts, and EA extracts of ADS024 and DSM7 did not affect proinflammatory cytokine secretion (TNFα, IL-1β, and MIP-1α) in toxin-treated human colonic explants from colorectal cancer patients (Supplementary Figures 1A–C).

## Discussion

This study is the first to explore the direct protective effects of ADS024 in *C. difficile* toxin-treated human colonic tissues and human epithelial cells. ADS024 filtrate can degrade toxin B. Both proteolytic ADS024 filtrate and non-proteolytic ADS024 EA extract protected against toxin B-mediated colonic injury. We discovered that the protective effects of ADS024 are mediated by the prevention of apoptosis.

A previous clinical study demonstrated that inhibition of toxin B, but not toxin A, is crucial for the clinical improvement of patients with CDI (Wilcox et al., 2017). ADS024 live bacteria secrete proteases, which can also be found in the filtrate. ADS024 filtrates generated from 30-min incubation of ADS024 bacterium caused partial degradation of toxin B, but not toxin A (Figure 5B). On the other hand, ADS024 filtrate generated from overnight incubation of ADS024 bacterium in brain-heart infusion (BHI) bacterial culture medium conferred additional capability to degrade both toxin A and toxin B, independent of the presence of *C. difficile* (O’Donnell et al., 2022). Indeed, we are unsure whether different bacterial enzymes degraded toxin A and toxin B or if extended incubation increased the enzyme concentration to degrade both toxins. The potential toxin B-degrading agent in the ADS024 filtrate should be above 50 kDa in molecular weight (Figure 5C). However, we could not further identify the exact toxin-degrading protease(s) amongst the 19 proteases (27–71 kDa) encoded by ADS024 (O’Donnell et al., 2022).

Based on our findings (Figure 5D), the ADS024 protease-mediated degradation of toxin B might be sufficient to inhibit toxin B-dependent apoptosis in human colonic epithelial cells and epithelial injury in fresh human colonic explants (Figures 1A, 2A, B). Similarly, the probiotic strain *Saccharomyces boulardii* produces a 54 kDa toxin-degrading protease and confers protection to the human colonic mucosa (Castagliuolo et al., 1999). Therefore, we believe that the ADS024 protease-dependent inhibition of toxin B effects might be mediated by decreased toxin levels.

In addition, ADS024 filtrate at 1–0.0001X effectively digested toxin B (Figure 5B), but ADS024 filtrate at 1–0.01X significantly reduced cell viability in human colonic epithelial cells (Supplementary Figure 2A). We speculate that ADS024-derived protease(s) is associated with cell viability inhibition. When the filtrate of ADS024 was diluted to 0.0001X, the cell viability inhibition became insignificant (Supplementary Figure 2A). Thus, the diluted ADS024 filtrate at 0.0001X was appropriate for mechanistic studies to address the anti-apoptotic mechanism in colonic epithelial cells (Figures 5, 6). Interestingly, the intestinal tissues tolerated ADS024-derived proteases as exposure to the highest concentration of ADS024 filtrate at 1X did not damage their epithelial structure (Figure 2A).

The proteolytic enzymes were eliminated during the isopropanol and ethyl acetate extraction process. The IPA extract of DSM7, but not ADS024, at 1X slightly reduced cell viability in the serum-starved human colonic epithelial cells (Supplementary Figure 2B). On the other hand, ADS024 EA extracts at 1–0.1X and DSM7 EA extract at 0.1X induced cell proliferation (Supplementary Figure 2C). Therefore, the secreted metabolites in ADS024 and DSM7 EA extracts may confer potential cytoprotective benefits to the human colonic epithelial cells.

Ethyl acetate extracts of ADS024 and DSM7 and filtrates of ADS024 possess anti-apoptotic effects *via* inhibition of caspase 3 cleavage (Figure 6C). Caspase 3 is a crucial molecule in the apoptosis pathway that mediates DNA fragmentation and cell morphological changes, a hallmark sign of apoptosis (Janicke et al., 1998). The anti-apoptotic metabolites of ADS024 and DSM7 were likely secreted products since EA extracts were generated from conditioned media, which did not contain bacterial cells. On the other hand, it is interesting to note that the cell-free supernatant and IPA extract of ADS024 inhibited *C. difficile* growth *in vitro* (O’Donnell et al., 2022). ADS024 possesses both anti-*C. difficile* and direct cytoprotective activities. However, we cannot identify the anti-*C. difficile* and cytoprotective factors in ADS024 because many biochemicals might be involved in these effects. Multiple factors are likely responsible for the anti-*C. difficile* and cytoprotective effects of ADS024. Additional investigations will be required to further characterize the respective functions of these protective factors.

There are a few cases of sepsis caused by probiotic *Lactobacillus* and fungemia caused by *Saccharomyces boulardii* (Land et al., 2005; Thygesen et al., 2012). Fecal microbiota transplantation was also associated with several deaths due to antibiotic-resistant bacteremia (DeFilipp et al., 2019). However, these complications are rare and should not impede the development of novel microbial approaches for treating CDI. Optimization for improving the efficacy and safety of microbial CDI therapies is in progress. We believe that SS-LBPs can be a safe adjunct prophylactic approach to prevent CDI recurrence (Spinler et al., 2016; Shen et al., 2017).

The identification of the toxin-degrading proteases and cytoprotective metabolites of ADS024 will be investigated further in the future. The identification of the important cytoprotective metabolites, which may involve synergism for efficacy, will require a lengthy and significant effort, and thus a significant limitation of this research is that it is not yet known what metabolites are responsible for the biological effects reported in the present manuscript.

In summary, ADS024-secreted metabolites demonstrated a protective effect against toxin B-mediated apoptosis in human colonic tissues and epithelial cells *via* the prevention of caspase 3-dependent apoptosis. The control strain DSM7 did not perform as well as ADS024 in these assays. The activity of ADS024 is mediated through toxin B-degrading activity and secretion of anti-apoptotic agent(s) against both toxins (Figure 7C). These findings support advanced studies of ADS024 in human testing and continued investigation as an SS-LBP candidate for the treatment and prevention of CDI.

## Data availability statement

The original contributions presented in this study are included in the article/Supplementary material, further inquiries can be directed to the corresponding author.

## Ethics statement

The studies involving human participants were reviewed and approved by UCLA IRB 12-001499. Written informed consent for participation was not required for this study in accordance with the national legislation and the institutional requirements.

## Author contributions

YX, AC, and BN: data acquisition. HF: production of *C. difficile* toxins. CP: critical revision of the manuscript. LC: study sponsor and critical revision of the manuscript. HK: study design, study supervision, data analysis, and writing of the manuscript. All authors contributed to the article and approved the submitted version.
