# Supplementary material for: ADS024, a Bacillus velezensis strain, protects human colonic epithelial cells against C. difficile toxin-mediated apoptosis
Source: Front Microbiol. 2023 Jan 10;13:1072534. doi: 10.3389/fmicb.2022.1072534 (PMC9873417; doi:10.3389/fmicb.2022.1072534)

Supplementary Table and Figure Legends:

Table S1

Baseline characteristics of fresh human colonic explants.

Figure S1

ADS024 and DSM7 products did not affect proinflammatory cytokine secretion in human colonic explants.

(A-C) Cytokine ELISA. TNFα, IL-1β, and MIP-1α levels in conditioned media of fresh human colonic explants after 6 hours of incubation. (A) ADS024 and DSM7 sterile filtrate did not affect cytokine secretion. (B) ADS024 and DSM7 IPA extracts did not affect cytokine secretion. (C) ADS024 and DSM7 EA extracts did not affect cytokine secretion. Results were pooled from samples from 10 patients. One-way ANOVA tests were used.

Figure S2

Human colonic epithelial cell viability data.

(A-C) Cell viability assays. (A) Serum-starved NCM460 cells were treated with PBS or 0.1% v/v of ADS024 and DSM7 FS from 1X to 0.0001X. (B) Serum-starved NCM460 cells were treated with PBS or 1% v/v of ADS024 and DSM7 IPA from 1X to 0.0001X. (C) Serum-starved NCM460 cells were treated with PBS or 1% v/v of ADS024 and DSM7 EA from 1X to 0.0001X. (A-C) After 48 hours, the treated cells were added with a 5% v/v MTS reagent and incubated for 15 minutes. The 490nm absorbance was read by a 96-well plate reader. An increase in absorbance indicated cell proliferation. A decrease in absorbance indicated a loss of cell viability. ADS024 FS 1-0.01X and DSM7 IPA extract 1X reduced cell viability. ADS024 EA extract at 1-0.1X and DSM EA extract at 0.1X increased cell proliferation. Student's t-tests were used for two-group comparisons. Treatment groups were compared with their respective control group.

Supplementary Table 1

Baseline characteristics of fresh human colonic tissues

12/3/2020
AB06
BS240763.3
Age = 86-90
Gender = male
Disease = adenocarcinoma
Disease location = right colon (12 cm from the proximal/terminal ileal margin)

12/30/2020
AB07
BS243544.6
Age: 71-75
Gender: Male
Disease: Adenocarcinoma
Disease location: Descending colon

1/14/2021
AB08/CDM01
BS245445.6
Age: 61-65yo
Gender: Female
Disease: Adenocarcinoma
Disease location: Cecum (procured sample is from ascending/transverse colon)

3/4/2021
AB09/CDM02
BS250780
Age: 56-60yo
Gender: Male
Disease: Moderately invasive adenocarcinoma arising from large tubular adenoma
Disease location: Proximal ascending colon

3/11/2021
AB10/CDM03
BS251379
Age: 71-75yo
Gender: Female
Disease: well to moderately differentiated adenocarcinoma
Disease location: ascending colon, near hepatic flexure

4/12/2021
AB11
BS254499
Age: 81-85yo
Gender: Male
Disease: Adenocarcinoma of descending colon
Disease location: descending colon

4/15/2021
AB12
BS254762
Age: 76-80 yo
Gender: Male
Disease: mass of ascending colon
Disease location: ascending colon

4/15/2021
AB13
BS254763
Age: 61-65yo
Gender: Male
Disease: Adenocarcinoma of terminal ileum, s/p neoadjuvant therapy
Disease location: ascending colon

5/13/2021
AB14
BS257042.1
Age: 61-65 yo
Gender: Male
Disease: mass of ascending colon (biopsy proven carcinoma)
Disease location: ascending colon

6/17/2021
AB15
BS259024
Age: 76-80 yo
Gender: Female
Disease: mass of ascending colon (biopsy proven adenocarcinoma)
Disease location: ascending colon

Supplementary Figure 1

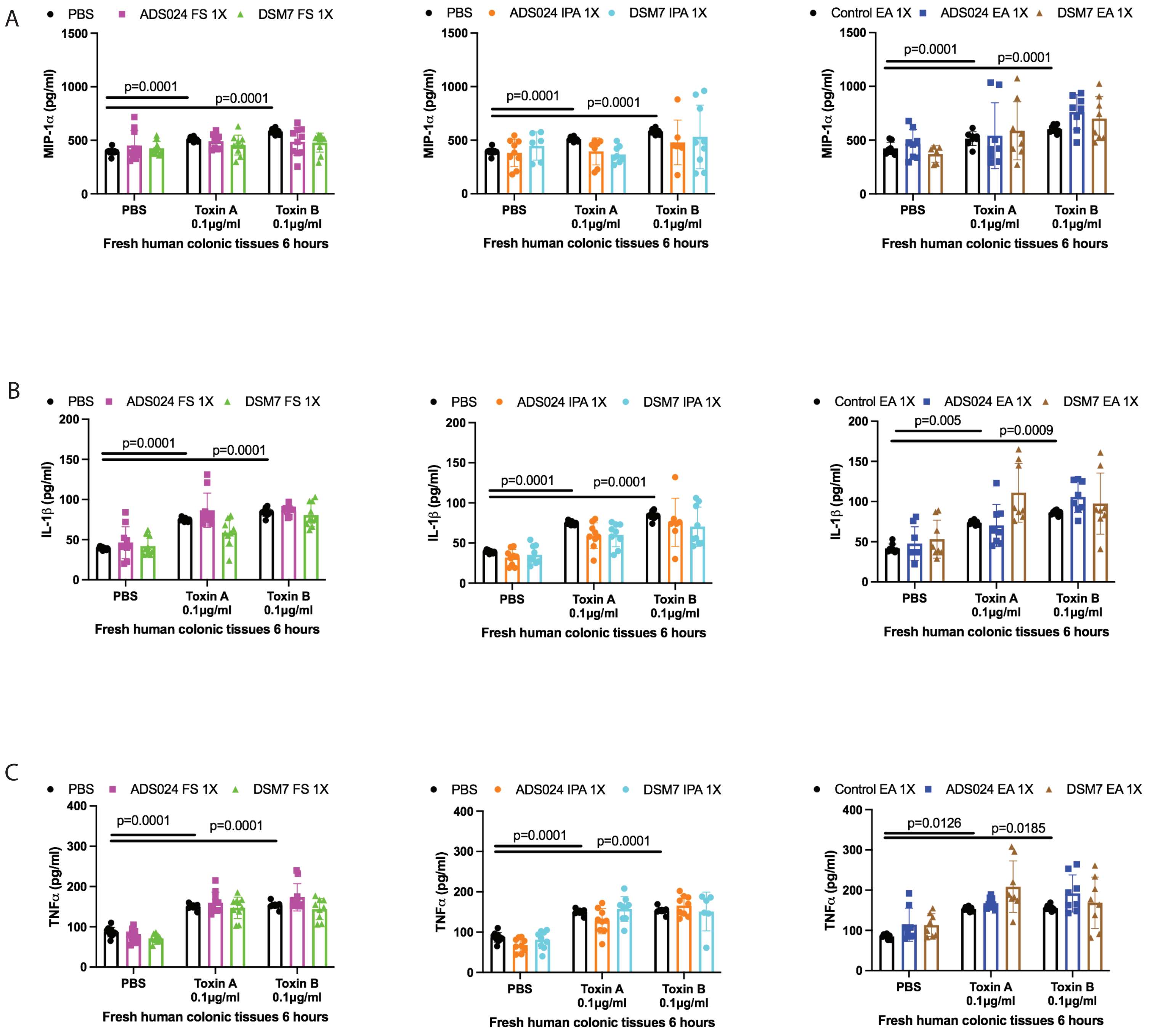

Supplementary Figure 2

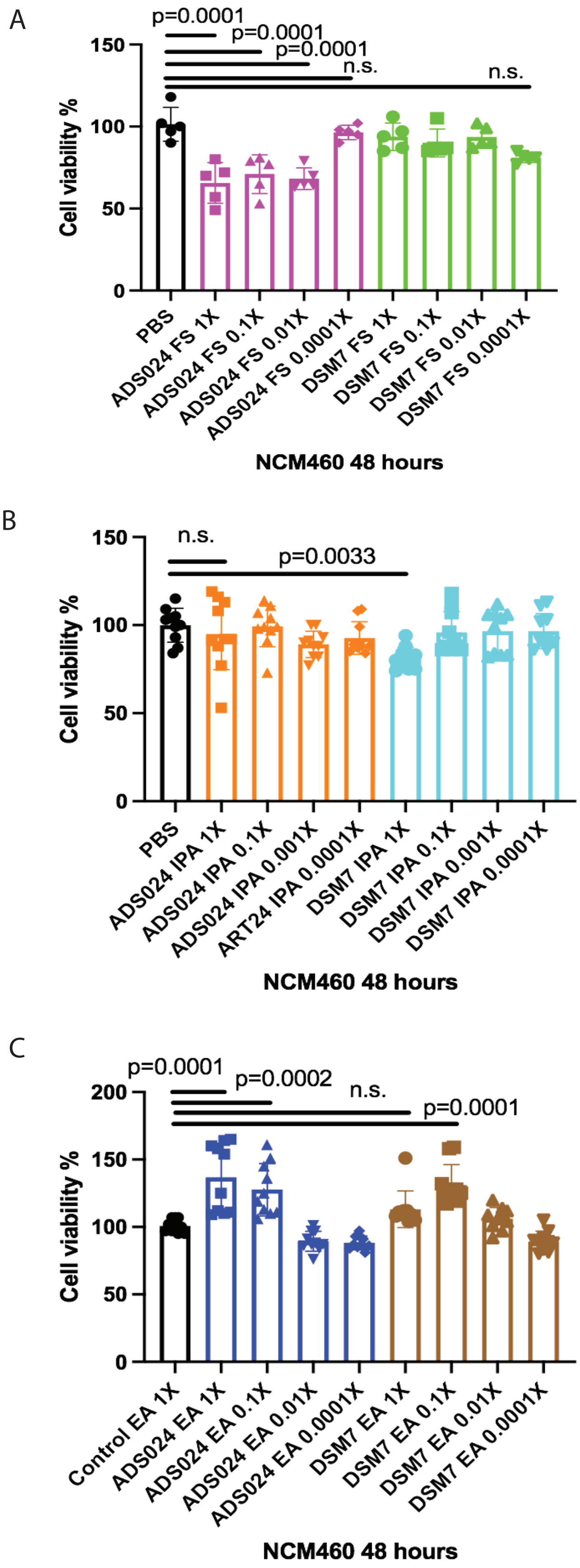

Supplement: Supplementary file 1 [file Data_Sheet_1.pdf]
